# Extracellular vesicles and chronic obstructive pulmonary disease (COPD): a systematic review

**DOI:** 10.1186/s12931-022-01984-0

**Published:** 2022-04-05

**Authors:** Nancy Gomez, Victoria James, David Onion, Lucy C. Fairclough

**Affiliations:** 1 School of Life Sciences, The University of Nottingham, Life Sciences Building, Nottingham, NG7 2RD UK; 2 School of Veterinary Medicine and Science, The University of Nottingham, Nottingham, NG7 2UH UK

**Keywords:** COPD, Chronic Obstructive Pulmonary Disease, Extracellular vesicles, Exovesicles

## Abstract

**Background:**

Chronic Obstructive Pulmonary Disease (COPD) is a common inflammatory disease of the airways characterized by irreversible airflow limitation, ranking the third highest cause of death worldwide. Extracellular vesicles (EVs) are important intercellular communication mediators released by cells into their extracellular environment with the capacity to transfer biological signals. EVs involved in COPD hold great potential to understand disease pathogenesis and identify important biomarkers. This systematic review aims to examine all available research on EVs in the pathogenesis and diagnosis of COPD to identify existing knowledge and support further research within the field.

**Methods:**

Publications were searched using PubMed and EMBASE with the search terms (Exosomes or extracellular vesicles or microvesicles or microparticles or ectosomes) AND (chronic obstructive pulmonary disease or COPD or emphysema or bronchitis).

**Results:**

Initial search yielded 512 papers of which 142 were manually selected for review and 43 were eligible for analyses. The studies were divided into groups according to the role of EVs in pathogenesis, EV origin and cargo, their role in COPD exacerbations and their diagnostic utility. EVs were found to be involved in the mechanism of pathogenesis of COPD, derived from various cell types, as well as containing modified levels of miRNAs. EVs also varied according to the pathophysiological status of disease, therefore presenting a possible method for COPD diagnosis and progress monitoring.

**Conclusion:**

The current findings show the limited but good quality research looking at the role of EVs in COPD, demonstrating the need for more studies to better define and provide further insight into the functional characteristics of EV in COPD pathogenesis.

**Supplementary Information:**

The online version contains supplementary material available at 10.1186/s12931-022-01984-0.

## Key messages

- What is the key question?
- How are extracellular vesicles (EVs) involved in Chronic Obstructive Pulmonary Disease (COPD)?
- What is the bottom line?
- EVs are involved in the mechanism of pathogenesis of COPD, derived from various cell types, with modified levels of cargo, and they present a possible method for COPD diagnosis and progress monitoring.
- Why read on?
- It is the first report of all available research on extracellular vesicles (EVs) in the pathogenesis and diagnosis of COPD and will be of particular interest and relevance to the medical and scientific readership of Respiratory Research as it identifies all existing research in this ever important and growing area of research.

## Introduction

Chronic Obstructive Pulmonary Disease (COPD) is a common inflammatory airway disease, affecting the airways, lung parenchyma and vasculature, and is characterized by irreversible airflow limitation. COPD has been found to affect about 10% of the population above 40 years of age and ranked the third highest cause of death worldwide [1]. It is estimated that 90% of all deaths from COPD can be linked to cigarette smoking [2]. Other risk factors include chronic exposure to biomass smoke, household indoor smoke and outdoor air pollution. Indeed, biomass fuels’ smoke has been identified as an independent risk factor leading to the development of COPD, particularly in low- and middle-income countries highly dependent on the use of biomass fuels [3]. COPD comprises of two diseases, emphysema and chronic bronchitis, whereby an abnormal inflammatory response in the lungs occurs after exposure to noxious particles or gases, leading to airway obstruction and emphysematous changes [4]. Symptoms of COPD are commonly chronic cough, excessive mucus production, air trapping, dynamic hyperinflation and shortness of breath upon physical exertion [1, 3, 5]. Indeed, COPD exhibits symptoms beyond the lung, with systemic manifestations, such as inflammation, and is often associated with other diseases, such as cardiovascular diseases and metabolic syndrome [6]. COPD may manifest frequent periods of exacerbation which is linked to increased airway and systemic inflammation and presents symptoms of breathlessness and sputum production that worsen acutely [7, 8]. COPD exacerbation is mainly common in patients with advanced COPD and has also been associated with viral or bacterial infections [9].

Extracellular vesicles (EVs) are important intercellular communication mediators released by cells into the extracellular environment [10]. EVs have the capacity to transfer biological signals between cells and as such, influence recipient cell function [11]. These signals are transmitted by various biomolecules including proteins, lipids, nucleic acids and sugars in phospholipid-enclosed vesicles that provide protection and allow for delivery to distal sites [11]. One of the main contents of EVs are microRNAs (miRNAs), small nucleic acids which play a significant role in the transmission of genetic information and in modulating protein synthesis, therefore affecting cellular functions. EVs can act in both an autocrine and paracrine manner, influencing a range of physiological and pathological functions of recipient cells [10–12]. EVs can be derived from most cell types and have been isolated from biological fluids like saliva, urine, nasal and bronchoalveolar lavage (BAL) fluid, amniotic fluid, breast milk, plasma, serum and seminal fluid [11]. They are classified into three groups based on size, biogenesis, and secretory component: (a) exosomes, (b) cellular microvesicles (microparticles/ectosomes), and (c) apoptotic bodies [11]. Exosomes are distinguished from other EV classes by their small size (approximately 50–100 nm), morphology, and their endosomal origin [11]. Exosomes are generated in multivesicular bodies (MVBs) in the form of intraluminal vesicles (ILVs), and once formed, the MVB can fuse with the plasma membrane to release its contents as exosomes [13]. Microvesicles (MVs) are larger in size (> 100 nm) and are produced by outward budding and fission of the cell membrane [11]. Apoptotic bodies (ApoBDs) are the largest of the EVs with a diameter of 1–5 μm and are generated from cells undergoing apoptosis [14]. In addition, both gram-negative and some gram-positive bacteria produce EVs, and as such may also play a role in pathogenesis of COPD as bacterial infections are linked to COPD and Acute Exacerbation of COPD (AECOPD) [9]. Bacterial EVs can also be found in indoor dust [15]. These EVs contain LPS on their surface and additional markers from their bacteria of origin and as such, inhalation of these EVs results in pulmonary inflammation, that when frequent, leads to emphysema [9].

EVs involved in COPD hold great potential to understand disease pathogenesis and identify important biomarkers. Here, a systematic review of all available research on EVs in the pathogenesis and diagnosis of COPD is presented to identify existing knowledge and support further research within the field.

## Methodology

### Sources and searches

This review was conducted with the implementation of the Preferred Reporting Items for Systematic Reviews and Meta-Analyses (PRISMA) guidelines. Publications were searched using PubMed and EMBASE for results up to 02 February 2022 with the search terms (exosomes or extracellular vesicles or microvesicles or microparticles or ectosomes) AND (chronic obstructive pulmonary disease or COPD or emphysema or bronchitis).

### Study selection 1

This systematic review was done to examine evidence on the role and function of EVs in the pathogenesis of COPD, with the hypothesis that EVs from various origins contribute to the pathogenesis of COPD. For this review, publications included were only primary research literature based on in vivo and in vitro human and animal studies that have been peer reviewed. Publications were screened by reviewing the full text of the articles, using predefined inclusion and exclusion criteria to first determine whether to include or exclude them (Table 1).

**Table 1:** Inclusion and exclusion criteria used for study selection

Inclusion criteria	Exclusion criteria
Research involving the extraction, identification, or production of EVs or their contents, such as DNA, miRNA, or protein	Research involving nanoparticles but not extracellular vesicles from a cell source
Researching involving COPD, emphysema, bronchitis or any disease closely related to COPD in the respiratory tract	Research involving lung disease but not specifically COPD
Isolation method of EVs included	Research involving treatment but not pathogenesis or diagnosis of COPD
	Non-original research paper, e.g. reviews, commentary, case report, etc.
	Articles published in a language other than English

### Study quality assessment

To assess the quality of the publications reporting on EVs and COPD, the studies were screened for components concerning the study of EVs and the diagnosis of COPD in study populations. The evaluation was done on publications pertaining to human studies only, allowing for evaluation on the study population based on size (score: n/3), small (n < 20) or large (n > 20), and number of control groups (score: n/2). Studies were scored according to size (n/3) using the following scale: small study populations = 1, a large study population with a small control group = 2, large study populations for all groups = 3. They were then scored for control groups (n/2), where no control group = 0, one control group = 1, and two or more control groups = 2. Studies were also assessed on the method of diagnosis of COPD (n/2), where diagnosis with spirometry only = 1 and diagnosis with GOLD criteria = 2.

Additionally, EV research is a relatively new and developing field of study, and as such criteria was set for experimental approach and techniques relating to EV studies [16]. Isolation of EVs (n/3) was scored as either 1-less preferable, 2-mid range, or 3-preferred techniques. Characterisation of EVs was also evaluated and ranked out of 4 marks. Together the total was 14 marks for all components added. This allowed for ranking from a score out of 1.0, where 1.0 indicated a high-quality study.

## Results

### Results of PRISMA statement evidence search and selection

**Fig. 1:**
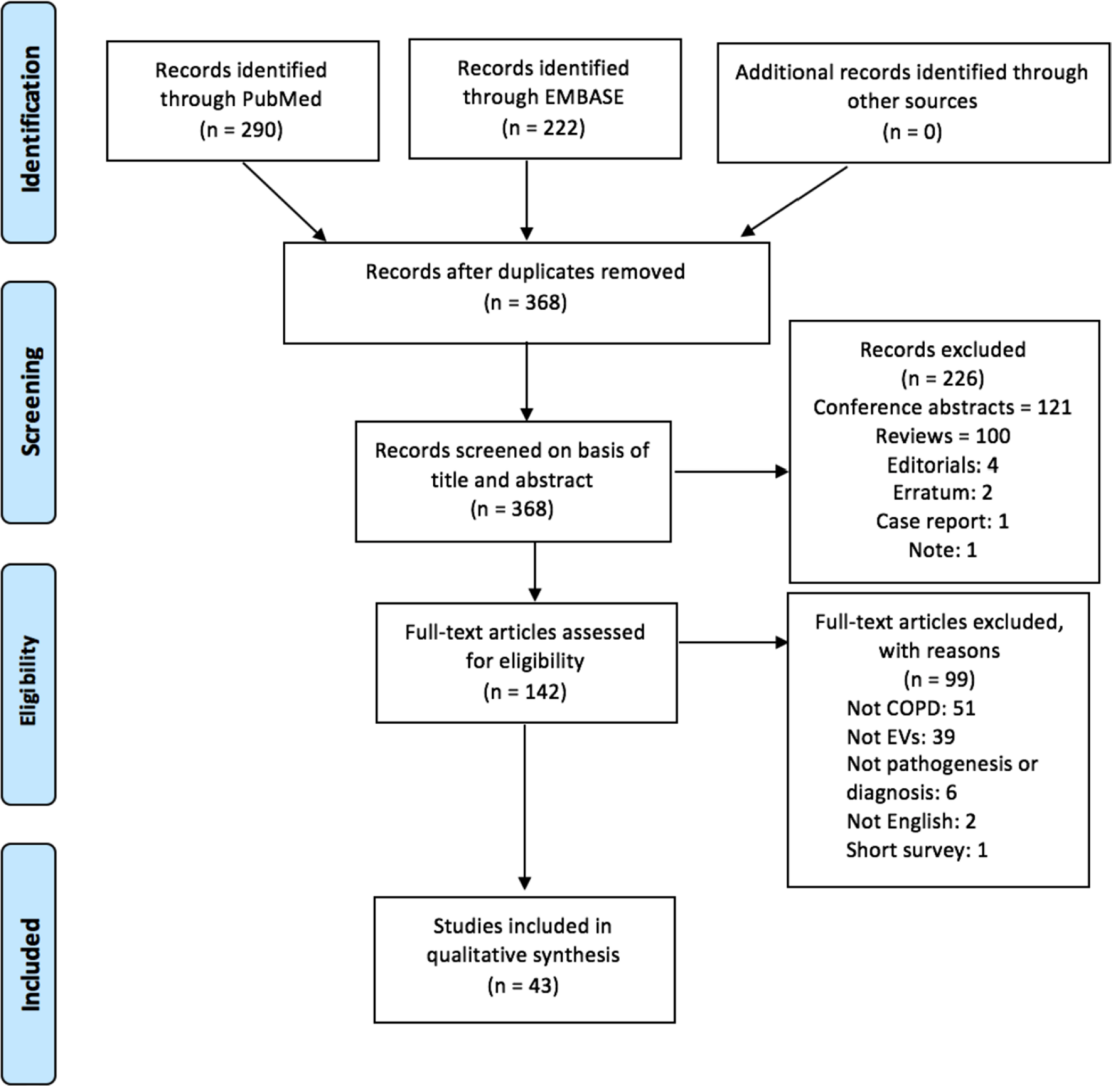
Summary of preferred reporting items for systematic reviews and meta-analyses (PRISMA) flow diagram

**Fig. 2:**
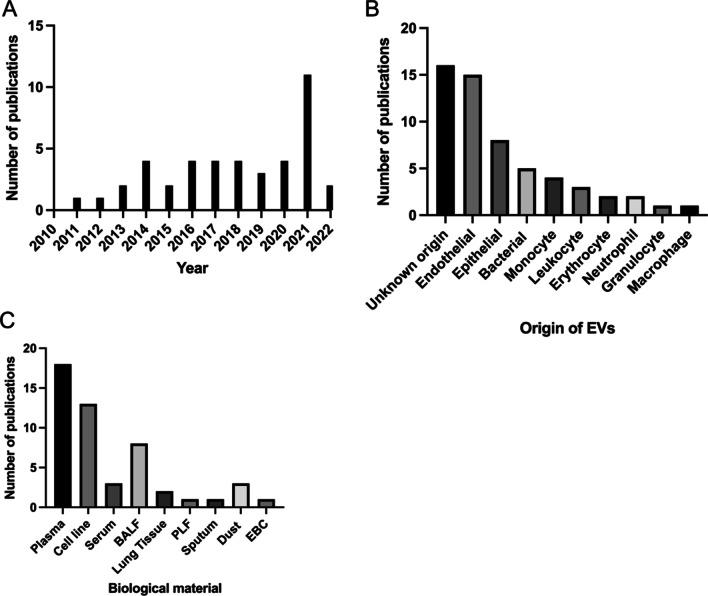
Results of the literature search. The graphs show the numer of publications (**A**) per year since the first publication in 2011, **B** according to the cell origin of EVs and **C** according to the biological material in which the EVs were studied. *BALF* broncoalveolar lavage fluid, *PLF* peritoneal lavage fluid, *EBC* exhaled breathe condensate

The EMBASE search identified 222 publications and the PubMed search identified 290 papers. Duplicates were removed (n = 144), leaving 368 papers whose titles and abstracts were screened for relevance to research topic. Articles were then screened on the basis of title and abstract to assess whether they reported EVs in COPD including bronchitis and emphysema, excluding 266 articles. The remaining 142 articles were fully reviewed for eligibility, and 43 papers fulfilled the criteria and were included in the present review. A detailed diagram of the review process can be seen below in Fig. 1. The first study was published in 2011 with the number of publications increasing each year and peaking with 11 publications in 2021 (Fig. 2A). The most common cell origin of the EVs studied were from endothelial cells, although some studies did not specify the origin (Fig. 2B). Additionally, the most common biological fluids from which EVs were studied were plasma, followed by cell lines (Fig. 2C).

### Mechanism of EVs in the pathogenesis of COPD

#### Endothelial EVs in COPD

Cigarette smoking (CS) causes stress and damage on the endothelial layer and induces the release of EVs from endothelial cells, therefore it is important to note the relationship of these EVs on the development of lung damage that may lead to COPD. In total 8 original research articles were identified that investigated endothelial EV (EEVs) levels in COPD (Table 2). Overall, the 8 studies [17–24] noted an elevation in CD31+/CD42b− or CD31+CD62E+ endothelial microparticles (EMPs) in their investigations. Six [18–21, 23, 24] of these studies were human studies, observing EMP levels in COPD patients and of the four studies, three studies compared EMPs in COPD patients with healthy smokers and non-smokers. One study was in vitro [17] and one was an animal study [22].

**Table 2:** Summary of studies on the mechanism of endothelial extracellular vesicles in COPD

Mechanism—endothelial cells				
Author, year [Ref.]	Title	Aim	Type	Conclusion
Takahashi et al., 2013 [17]	Differences in the released endothelial microparticle subtypes between pulmonary microvascular endothelial cells and aortic endothelial cells in vitro	Evaluate the effects of common stimuli involved in COPD on endothelial microparticles (EMPs) released. Investigate whether increased circulating EMP subtypes reflect the degree and site of endothelial injury in COPD patients	In vitro	H_2_O_2_ and cigarette smoke extract (CSE) induced apoptosis, resulting in the release of PECAM EMPs from pulmonary ECs and MCAM EMPs from both pulmonary and aortic EC types. TNF-a stimulation resulted in EC activation, resulting in the upregulation of E-selectin, a mechanism that occurs during COPD exacerbation. Thus, EMP subtypes reflect differences among stimuli and site of injury in COPD mechanism
Strulovici-Barel et al., 2016 [18]	Persistence of circulating endothelial microparticles in COPD despite smoking cessation	Investigate whether elevated levels of circulating apoptotic EMPs persists in COPD smokers following smoking cessation, reflecting continuous lung endothelial injury that persists even after the stress of smoking is removed	Ex vivo Human	Total pulmonary capillary EMP levels were highest in healthy smokers, followed by COPD smokers, when compared to non-smokers, with 48% of healthy smokers and 45% of COPD smokers showing increased levels of apoptotic EMPs. This suggests active pulmonary capillary apoptosis ongoing in both healthy and COPD smokers that persisted even after they stopped smoking following their baseline assessment
Thomashow et al., 2013 [19]	Endothelial microparticles in mild chronic obstructive pulmonary disease and emphysema	Examine the relationships of circulating levels of EMPs with COPD	Ex vivo Human	CD31+ EMPs were elevated in COPD and were positively related to percent emphysema. Additionally, CD62E+ EMPs were elevated in severe COPD and with hyperinflation. These cellular markers may implicate endothelial apoptosis in the pathogenesis of COPD and emphysema
Garcia-Lucio et al., 2018 [20]	Imbalance between endothelial damage and repair capacity in chronic obstructive pulmonary disease	Investigate whether COPD patients have an imbalance between EMPs to PCs (progenitor cells) compared to non-smokers and current smokers. Evaluate the effect of cigarette smoke on these circulating markers	Ex vivo Human	COPD patients presented a significantly disturbed ratio of elevated circulating apoptotic EMP levels with reduced bone marrow-derived PC numbers, reflecting an imbalance between endothelial damage and reduced repair capacity
Barak et al., 2017 [21]	Disturbed blood flow worsens endothelial dysfunction in moderate-severe chronic obstructive pulmonary disease	To test whether oscillatory shear stress further exacerbates endothelial dysfunction in patients with moderate-severe COPD and to observe any potential link between chronic hypoxemia and EMPs in COPD	In vivo/ex vivo Human	In moderate-severe COPD patients, acutely disturbed blood flow further deteriorates endothelial dysfunction that is compounded with increases in circulating MPs indicative of endothelial apoptosis (CD31+/CD41b−), and is of greater consequence given the already impaired vasculature of this population
Liu et al., 2014 [22]	Circulating endothelial microparticles involved in lung function decline in a rat exposed in cigarette smoke maybe from apoptotic pulmonary capillary endothelial cells	Investigate if the number of EMPs is elevated in rats exposed in cigarette smoke, and whether the elevated EMPs are derived from pulmonary capillaries	In vivo Mice	Exposure of rats to CS resulted in high levels of circulating CD42b/CD31+ EMPs (cEMPs), which increased with an increase in time of exposure. High levels of CD42b/CD31+ cEMPs reflected the decline of small airway function indirectly in early COPD and would be useful for evaluating the degree of COPD progression
Nieri et al., 2021 [23]	Circulating extracellular vesicles are associated with disease severity and interleukin-6 levels in COPD: a Pilot study	Analyse endothelial-(E) and monocyte-derived (M) EV levels in COPD patients grouped according to the 2011 GOLD classification and analyse the relationship between EV and plasmatic markers of inflammation	Ex vivo Human	Circulating endothelial- and monocyte-derived extracellular vesicles increase along with COPD severity. The relationship among EEV and IL-6 suggests a biological link between inflammation and endothelial activation/damage
Lascano et al*.*, 2021 [24]	Association of systemic endothelial-derived and platelet-derived microparticles with clinical outcomes in chronic obstructive pulmonary disease	Analyse whether eMPs and pMPs are associated with COPD status and/or severity	Ex vivo Human	Most MPs measured do not correlate significantly with COPD status, COPD severity, or exacerbations in our cohort. The apoptotic eMP 62E+/eMP 31+ ratio may be a useful marker of early endothelium apoptosis and early recognition of the disease process. Platelet activation assessed by pMP 41+31+ increases with disease severity and may be an important feature for stage 4 COPD patients

*EMPs* endothelial microparticles, *PECAM* CD31(+)/CD41(−) microparticles, *EC* endothelial cells, *MCAM* CD146(+) microparticles

The increased levels of CD31+/42b− EMPs suggested for all 8 studies that there was active endothelial apoptosis and endothelial damage. However, four studies [18, 20, 22, 23] made additional observations. Strulovici-Barel [18] noted that upon smoking cessation, healthy smokers had a significant decrease in total EMP levels after 12 months, compared to healthy smokers who continued smoking, but that for COPD patients, the EMP levels had no significant change for patients who quit smoking compared to those that continued to smoke. Garcia-Lucio et al. [20] observed a significantly elevated EMP level for COPD patients and healthy smokers when compared to healthy non-smokers, noting also a reduced number of progenitor cells (PCs) for the COPD patients which would reflect an imbalance between endothelial damage and a reduced repair capacity. Liu et al. [22] observed, in an animal model of COPD, that the elevated levels of CD31+/42b− EMPs increased with an increased time of CS exposure. Nieri et al. [23] observed a direct relationship between EEVs and IL-6, suggesting there is release of EEVs upon an inflammatory stimulus.

Six [18–21, 23, 24] of the studies were assessed for quality, given as they were human studies. The articles ranged from a score of 0.57 for the lowest quality to 0.86 for two studies with higher quality. All the studies included for assessment lost marks in the EV isolation method, but scored high for EV characterisation. The study that scored the lowest had a large study population but did not include any control group (Additional file 1: Table S1).

#### EVs from other cell types in COPD

Other cell types that play a role in the pathogenesis of COPD are also known to release EVs. Eleven studies [23, 25–34] observed EVs from other cell types involved in COPD (Table 3). Seven of the studies [25, 26, 28, 30–33] investigated EVs from epithelial cells, two studies [27, 34] observed EVs from neutrophils, one study [29] examined microparticles from T lymphocytes and one study [30] did not specify the cells types EVs were derived from. Six studies investigating EVs from epithelial cells carried out in vitro studies, and three of the studies carried out further analysis in vivo in mice only [26] or in vivo mice and ex vivo human [28]. The six studies noted that CS triggered and affected the release of EVs from epithelial cells, and of these studies, four [26, 28, 32, 33] made additional observations in terms of the cargo in the EVs. Genschemer et al. [27] observed EVs from neutrophils and carried out in vitro studies using cell lines and in vivo mice and ex vivo human studies. The study concluded that exosomes released from neutrophils carried neutrophil elastase (NE) and were found to bind to the extracellular matrix (ECM), leading to emphysema. Margaroli et al. [34] also studied neutrophil-derived EVs and EV-bound NE in mediating emphysema. Qiu et al. [29] observed that T lymphocytes microparticles (TLMPs) were significantly upregulated in COPD patients compared to healthy volunteers and further noted that CD4+ and CD8+ TLMPs reduced cell viability and induced production of inflammatory cytokines. Nieri et al. [23] observed an increase in monocyte-derived EVs with COPD severity. Zou et al. [30] noted significantly increased levels of IL-1B-containing exosomes in the bronchoalveolar lavage from mice with emphysema, but did not specify the cells the exosomes were derived from.

**Table 3:** Summary of studies on the mechanism of EVs derived from other cell types in COPD

Mechanism—other cell types				
Author, year [Ref.]	Title	Aim	Type	Conclusion
Benedikter et al., 2017 [25]	Cigarette smoke extract induced exosome release is mediated by depletion of exofacial thiols and can be inhibited by thiol-antioxidant	Investigate whether oxidative components of CSE are responsible for EV release and whether this could be prevented using the thiol antioxidants *N*-acetyl-l-cysteine (NAC) or glutathione (GSH)	In vitro	CSE exposure enhances the exosome release by airway epithelial cells (AEC) and this is mediated by thiol-reactive compounds like carbonyl acrolein, which may act by depleting extracellular free thiols
Moon et al., 2014 [26]	CCN1 secretion and cleavage regulate the lung epithelial cell functions after cigarette smoke	Investigate whether CCN1 is a potentially crucial factor for the pathogenesis of CS-induced emphysema	In vitro In vivo *Mice*	CS enhanced the release of exosomes containing full-length CCN1 (flCCN1) from lung epithelial cells. Exosome-mediated secretion of flCCN1 triggers inflammatory responses by mediating IL-8 release to distant portions of the lungs and subsequent neutrophil recruitment. Additionally, cleaved CCN1 (cCCN1) were generated from exosome-enriched CCN1 via secreted plasmin and promoted emphysematous changes
Genschmer et al., 2019 [27]	Activated PMN exosomes: pathogenic entities causing matrix destruction and disease in the lung	Investigate whether neutrophil elastase (NE) exists in exosomal form and whether such exosomes might bypass a1AT and contribute to inflammatory lung disease	In vitro Ex vivo human In vivo Mice	NE exists in an active, substrate-accessible form when associated with exosomes from activated PMN (polymorphonuclear leukocytes, i.e. neutrophils) and is resistant to a1AT. Activated PMN exosomes bind ECM via MAC-1 and degrade ECM via NE. CD66b+/NE+ PMN exosomes cause emphysema when administered to mice and when residing in COPD patients
Feller et al., 2018 [28]	Cigarette smoke-induced pulmonary inflammation becomes systemic by circulating extracellular vesicles containing Wnt5a and inflammatory cytokines	Demonstrate a potential mechanism for the systemic nature of COPD	In vitro Ex vivo human In vivo Mice	CS triggers release of EVs carrying pro-inflammatory cytokines and inflammation inducer Wnt5a, in turn triggering systemic inflammation and thus making COPD a complex disease that is hard to control
Qiu et al., 2020 [29]	Increased airway T lymphocyte microparticles in chronic obstructive pulmonary disease induces airway epithelial injury	Examine T lymphocyte microparticles (TLMP) subpopulations in BALF of patients with COPD and and explore the effects of MPs derived from different T cell subpopulations on airway epithelium	Ex vivo Human	The numbers of MPs derived from T lymphocytes in BALF were significantly upregulated in COPD patients compared with healthy volunteers. Isolated CD4+ and CD8+ TLMPs reduced cell viability and induced significant production of inflammatory cytokines including IL-6, MCP-1, MCP-2, MMP-9 and TNF-α in HBEs, while the levels of anti-inflammatory cytokine IL-10 were decreased. TLMPs in the airways may lead to airway epithelial injury and inflammation and serve essential roles in the pathophysiology of COPD
Zou et al., 2021 [30]	Release and actions of inflammatory exosomes in pulmonary emphysema: potential therapeutic target of acupuncture	Investigate if exosome-mediated release of NLRP3 inflammasome products instigates the inflammatory response in the lung during emphysema	In vivo Mice	NLRP3 inflammasome activation and associated inflammatory exosome release are critically implicated in the development of inflammation during PPE-induced emphysema
Wang et al., 2021 [31]	Cigarette smoke extract-treated airway epithelial cells-derived exosomes promote M1 macrophage polarization in chronic obstructive pulmonary disease	Investigate whether the exosomes derived from CSE-treated AECs regulate macrophage polarization and subsequently affect the progression of COPD by modulating TREM-1 expression	In vitro	Exosomes derived from CSE-treated AECs aggravate CS-induced lung inflammation and tissue injury in mice, which is associated with the promotion of M1 macrophage polarization by these exosomes through upregulation of TREM-1 expression
Song et al., 2021 [32]	Exosomal lncRNA TCONS_00064356 derived from injured alveolar epithelial type II cells affects the biological characteristics of mesenchymal stem cells	Investigate whether injured alveolar cells communicate with MSCs via secretion of exosomes and investigate the role of exosomal lncRNAs derived from injured alveolar cells to identify novel therapeutic targets for COPD	In vitro	Injured AEC-II cells can affect the biological characteristics of MSCs via secretion of exosomes and the dysregulated exosomal lncRNAs that may be involved in this process were screened out
Xia et al., 2022 [33]	The aberrant cross-talk of epithelium-macrophages via METTL3-regulated extracellular vesicle miR-93 in smoking-induced emphysema	Assess the role of EV miR-93 in bronchial epithelium exposed to cigarette smoke and the cross-talk between these cells and macrophages in smoking-induced emphysema	In vitro	CS exposure induces elevation of METTL3-promoted miR-93 maturation, and miR-93 is transferred from bronchial epithelial cells into macrophages by EVs. In macrophages, miR-93 activates the JNK pathway by targeting DUSP2, which increases the levels of MMP9 and MMP12, inducing elastin degradation. Therefore, CS induces emphysema by a mechanism in which METTL3-mediated EV miR-93 via m6A is involved in aberrant cross-talk of lung epithelial cells and macrophages
Margaroli et al., 2022 [34]	A novel in vivo model for extracellular vesicle-induced emphysema	Develop a mouse-to-mouse EV-transfer model to expand on neutrophil-derived EVs and further explore discrete disease-related mechanisms	In vivo Mice	This study highlights a rapid, novel neutrophil driven mechanism of emphysema mediated by mouse neutrophil derived EV-bound NE. EVs from in vivo LPS activated mouse neutrophils induced COPD-like disease in naive recipients through an alpha-1 antitrypsin resistant, NE-dependent mechanism
Nieri et al., 2021 [23]	Circulating extracellular vesicles are associated with disease severity and interleukin-6 levels in COPD: a Pilot study	Analyse endothelial-(E) and monocyte-derived (M) EV levels in COPD patients grouped according to the 2011 GOLD classification and analyse the relationship between EV and plasmatic markers of inflammation	Ex vivo Human	Circulating endothelial- and monocyte-derived extracellular vesicles increase along with COPD severity. The relationship among EEV and IL-6 suggests a biological link between inflammation and endothelial activation/damage

Three studies were assessed, where two [27, 28] studies scored 0.64 and one study scored 0.79. The two studies scored poorly in terms of study population, where both had small group sizes and only one control, and one study [28] did not include how COPD patients were diagnosed with the condition (Additional file 1: Table S2). The third study scored [29] had good population size and EV characterisation techniques.

#### EVs containing microRNA in COPD

EVs carry various biomolecules and of particular interest are nucleic acids including mRNAs, miRNAs and non-coding RNAs and DNA sequences. Interestingly, some EVs have the ability to export miRNA outside cells and affect gene expression in distance cells, thereby inducing phenotypic changes [10, 35]. Four studies [36–39] investigated whether EVs containing miRNA could influence manifestations of COPD (Table 4). These studies all conducted in vitro studies, with three of the studies [36, 37, 39] carrying out additional human and animal studies. All four studies observed that CS caused changes in the miRNA levels in EVs. Two studies [36, 37] observed that CS modified the levels of miR-21 carried by EVs. He et al. [36] noted that CS reduced levels of miR-21 of EVs derived from BEAS-2B cells but that EVs obtained from the serum of COPD patients carried significantly higher levels of miR-21. Xu et al. [37] concluded that CS exposure increased miR-21 levels in exosomes from human bronchial epithelial cells (HBECs). Two studies [38, 39] observed that CS modified the miRNA components in EVs. Fujita et al. [38] noted that EVs from HBECs treated with CS had distinct and varying levels of 8 miRNAs, which had either increased levels or decreased levels when compared to the non-treated group. Together, the studies suggest that the CS-induced changes in miRNA cargo of EVs result in myofibroblast differentiation [37, 38], inference of efferocytosis [39] or polarization of macrophages to M2 [36], which are all characteristic of the pathogenesis in COPD.

**Table 4:** Summary of studies on EVs containing miRNA in COPD

Mechanism—microRNA				
Author, year [Ref.]	Title	Aim	Type	Conclusion
He et al., 2019 [36]	Bronchial epithelial cells extracellular vesicles ameliorate epithelial–mesenchymal transition in COPD pathogenesis by alleviating M2 macrophage polarization	Investigate whether EVs could influence the occurrence of inflammatory lung disease (in particular COPD) through contained microRNAs	In vitro Ex vivo human In vivo Mice	EVs found in the serum contained significantly higher levels of miR-21 in COPD patients than healthy people. CS was found to reduce levels of miR-21 in EVs of BEAS-2B cells at 1%, 3% and 5% when compared to 0%. Macrophages were then found to uptake these EVs, resulting in polarization into M2 phenotype which negatively correlated with lung function of mouse COPD models
Xu et al., 2018 [37]	Exosomal microRNA-21 derived from bronchial epithelial cells is involved in aberrant epithelium-fibroblast cross-talk in COPD induced by cigarette smoking	Evaluate the expression of miR-21 in exosomes derived from bronchial epithelial cells exposed to CSE and investigate the mechanism for exosomal miR-21 in airway remodeling	In vitro Ex vivo Human In vivo Mice	CS exposure induces increases of miR-21 levels transferred by exosomes from human bronchial epithelial (HBE) cells into bronchial fibroblast cells, promoting myofibroblast differentiation by increases of HIF-1a transcriptional activity
Fujita et al., 2015 [38]	Suppression of autophagy by extracellular vesicles promotes myofibrobasts differentiation in COPD pathogenesis	Investigate an EV-mediated intercellular communication mechanism between primary human bronchial epithelial cells (HBECs) and lung fibroblasts (LFs)	In vitro	CSE-induced HBEC derived EVs had modified components, 8 miRNAs including miR-210, when compared to non-treated HBEC-derived EVs. HBEC-derived EV miR-210 caused significant increase in collagen type I and a-SMA expression in lung fibroblasts (LFs) which are characteristic of myofibroblast differentiation via EVs
Serban et al., 2016 [39]	Structural and functional characterization of endothelial microparticles released by cigarette smoke	Investigate the role of signaling of pathways typically involved in endothelial apoptosis in EMPs release and the role of CS-induced EMPs and their impact on specialized phagocytes	In vitro Ex vivo Human In vivo/ex vivo mice	CS, via aSMase, releases circulating EMPs with distinct microRNA cargo. CS-induced apoptotic and exosomal EMPs carry ceramides and specific miRNAs in circulation and result in interference of efferocytosis

Three [36, 37, 39] of the four studies were assessed for quality and scores ranged from 0.64 to 0.93 for the highest quality paper (Additional file 1: Table S3). The study with the highest score had population groups above n = 20 and two additional control groups (non-smokers without COPD and smokers without COPD), and diagnosed COPD patients according to GOLD criteria. Additionally, EV isolation methods included centrifugation plus an exosome precipitation solution kit. Finally, characterisation of EVs for this study was done via nanoparticle tracking analysis, TEM and western blotting.

#### Bacterial EVs and COPD

EVs are known to also be released from all gram-negative and some gram-positive bacteria, which carry a wide variety of molecules such as proteins, lipids, DNA, and RNA, but additionally harbour various virulence factors, such as LPS, and as a result, may have several physiological and pathological functions in bacteria-host interactions [9, 40]. Additionally, studies have demonstrated the presence of bacteria-derived EVs in indoor dust that has been collected from beds in apartments [15].

Four studies [41–44] investigated whether bacteria-derived EVs were related to COPD (Table 5). Three research articles [42–44] identified carried out ex vivo human studies and one study [41] was done in vivo using a mice model and additionally in vitro assays. Kim et al. [41] determined that EVs derived from bacteria resulted in emphysema due to neutrophilic inflammation. Kim et al. [42] showed that bacterial EVs differed in the lungs of COPD patients when compared to non-smokers and healthy smokers. The third study [43] observed that IgG levels against bacterial EVs collected from dust were highest in COPD patients than in control subject. Yang et al. [44] followed from this study to determine the specific bacterial EVs affecting COPD in indoor dust, such as *S. aureus*, *A. baumannii*, *E. cloacae* and *P. aeruginosa*. As a result, all together the studies show that bacteria-derived EVs whether found in lungs or inhaled from the environment were distinct in COPD patients than in control groups and could result in pathogenesis of COPD.

**Table 5:** Summary of studies on bacterial EVs and COPD

Mechanism—bacterial EVs				
Author, year [Ref.]	Title	Aim	Type	Conclusion
Kim et al., 2015 [41]	Extracellular vesicles derived from gram-negative bacteria, such as *Escherichia coli*, induce emphysema mainly via IL-17A-mediated neutrophilic inflammation	Investigate whether *E. coli* EVs are casually related to the pathogenesis of emphysema, and determine the immunologic mechanisms of emphysema induced by *E. coli* EVs	In vivo Mice In vitro	Airway exposure of EVs derived from Gram-negative bacteria, especially *E. coli*, can induce neutrophilic inflammation and thereby emphysema mainly in an IL-17A–dependent manner. TLR4 signaling is important in the uptake of *E. coli* EVs and the production of proinflammatory cytokines induced by interaction with LPS on *E. coli* EVs
Kim et al., 2017 [42]	The microbiome of the lung and its extracellular vesicles in nonsmokers, healthy smokers and COPD patients	Investigate whether the microbiome of lung EVs might have distinct characteristics depending on the presence of COPD and smoking status	Ex vivo Human	Bacteria-derived EVs have distinctive characteristics in the lungs of non-smokers, healthy smokers and patients with COPD. According to the Shannon index, non-smokers demonstrated most diversity in lung tissue compared to COPD patients which were least diverse. Also, diversity index for lung EVs showed most diversity in COPD patients and least in the non-smoker group. The Simpson index was highest in COPD group, indicative of dominant organisms
Kim et al., 2016 [43]	IgG sensitization to extracellular vesicles in indoor dust is closely associated with the prevalence of non-eosinophilic asthma, COPD, and lung cancer	To evaluate whether sensitization to indoor dust EVs is a risk for the development of COPD To determine whether serum antibodies against dust EVs associate with the increased risk of COPD	Ex vivo Human	Serum anti-dust EV IgG levels were significantly higher in patients with COPD than in the control subjects. Thus, IgG sensitization to dust EVs may increase the risk of COPD expression and/or development, providing an insight into the pathogenesis of COPD
Yang et al., 2020 [44]	Lung disease diagnostic model through IgG sensitization to microbial extracellular vesicles	To investigate whether exposure to bacterial EVs in indoor dust might be associated with the risk of asthma, COPD and lung cancer	Ex vivo Human	The specific bacterial EVs affecting pulmonary diseases in indoor dust, such as *S. aureus*, *A. baumannii*, *E. cloacae* and *P. aeruginosa*. Anti-core indoor dust bacterial EV IgG, IgG1 and IgG4 antibodies titres in serum were significantly higher in patients with COPD compared to the healthy control group

Three [42–44] of the studies were assessed and scores ranged from 0.50 to 0.64. Breakdown of their assessment can be noted in Additional file 1: Table S4. The studies missed marks in all categories of assessment, leading to a lower score.

### EVs and exacerbation of COPD

COPD is often marked by periods of exacerbation, whereby there is increased airway and systemic inflammation, and symptoms such as breathlessness and sputum production worsen and could lead to hospitalization [8]. Four studies [45–48] investigated whether EVs were involved in occurrences of COPD exacerbation (Table 6). Three studies [45, 47, 48] compared EV levels in stable and exacerbated COPD patients. All three studies noted elevated levels of EVs in patients with exacerbated COPD, and each carried out additional observations in their studies. Tan et al. [47] primarily focused on levels of exosomes (CD9 exosomes) and noted the highest levels in patients with acute exacerbation of COPD compared to stable COPD patients and healthy controls. The other two studies [45, 48] observed significantly elevated levels of CD62E+ (E-selectin) microparticles (MPs) in exacerbated COPD patients, with one [48] of the studies additionally observing CD41+, CD42a+ and CD14+ MPs levels in their study.

**Table 6:** Summary of studies on EVs in COPD exacerbation

COPD exacerbation				
Author, year [Ref.]	Title	Aim	Type	Conclusion
Takahashi et al., 2012 [45]	Increased circulating endothelial microparticles in COPD patients: a potential biomarker for COPD exacerbation susceptibility	To compare EMP numbers in stable COPD patients with those during and after exacerbation	Ex vivo Human	VE-cadherin (CD144 +), PECAM (CD31+/41−) and E-selectin (CD62E+) EMPs of pulmonary capillary origin were significantly more numerous in the stable COPD patients than in the healthy non-COPD volunteers, with further increase in the exacerbated phase. Baseline E-selectin EMP levels were significantly higher in COPD patients with frequent exacerbations than patients without, indicative of endothelial damage during exacerbation
Eltom et al., 2014 [46]	Respiratory infections cause the release of extracellular vesicles: implication in exacerbation of asthma/COPD	Investigate whether respiratory infections cause the release of EVs in the airway and that the raised ATP levels, present in respiratory disease, triggers the release of IL-1b/IL-18, neutrophilia and subsequent disease exacerbations	In vitro In vivo/ex vivo Mice Ex vivo *Human*	Respiratory infections, bacterial and viral, can trigger the release of functional EVs in mice and man. Upon ATP activation, the EVs released IL-1b and IL-18 in a P2X7/caspase-1 axis dependent manner resulting in exacerbated neutrophilia
Tan et al., 2017 [47]	Elevated levels of circulating exosomes in COPD patients are associated with systemic inflammation	Investigate whether the levels of circulating exosomes (CD9+ microvesicles) are abnormally elevated in individuals who experienced acute exacerbations of COPD (AECOPD) and whether exosomes are associated with systemic inflammation	Ex vivo Human	Exosomes (CD9 microvesicles) are elevated in COPD and AECOPD. Level of exosomes correlated with an increase in plasma CRP, sTNFR1 and IL-6, which are well-established markers of systemic inflammation and associated with COPD, with AECOPD group having the highest elevation when compared to sCOPD and healthy controls
Tokes-Fuzesi et al., 2018 [48]	Role of microparticles derived from monocytes, endothelial cells and platelets in the exacerbation of COPD	Measure EMPs and other cell-derived circulating MPs in stable and exacerbated COPD patients	Ex vivo Human	Highly elevated MP levels were found in COPD patients compared to controls, and in particular, CD62E+, CD41+, CD42a+ and CD14+ MPs were significantly increased in exacerbated COPD versus stable COPD, indicative of endothelial activity and vascular injury in the lungs. CD62E+, CD42a+ and CD14+ MPs correlated inversely with FEV1/FVC

The fourth study [46] identified carried out in vitro, in vivo mice and ex vivo human studies, focusing on the cargo of EVs that may result in COPD exacerbation. This study observed that EVs released during respiratory infections carried and released IL-1b and IL-18, suggesting a mechanism that results in disease exacerbation.

Three [45, 47, 48] of the studies were assessed for quality and scores ranged from 0.79 to 0.86 (Additional file 1: Table S5). All three studies scored low (1/3) in the EV isolation method, where precipitation was the only method used. The lowest scoring study (0.79) had a small sample size of study population when compared to the other two studies.

### EVs in COPD diagnosis

According to the Global Initiative for Chronic Obstructive Lung Disease (GOLD), the current diagnosis of COPD is based on three features including spirometry, observation of symptoms and a significant exposure to toxic stimuli [49]. Twelve studies [50–61] investigated the use of EVs as a more effective and accurate diagnostic tool for COPD (Table 7). All twelve studies involved human studies, with one study [53] carrying out additional investigation in vitro. Seven studies observed EV levels and five studies observed the cargo in the EVs. Six studies [50, 52, 54, 56, 58] noted elevated levels of EVs in COPD patients, observing specific microparticles. One study [50] observed significantly elevated levels of CD31+ MPs in the sputum of COPD patients that negatively correlated with forced expiratory volume in 1 s (FEV1). Another study [52] showed that elevated levels of CD62E+ MPs in plasma of COPD patients had significant negative correlations with FEV1 changes. The third study [54] identified a series of plasma EVs (CD45, CD28, CTLA4, TNF-R-II, and CD16) highly expressed in patients with COPD. Another study [51] noted elevated levels of EMPs with apoptotic characteristics in smokers with reduced DL_CO_, indicative of early lung destruction. Soni et al. [56] noted that BALF neutrophil MVs significantly correlated with clinically relevant disease severity indexes. Bazzan et al. [58] observed increased levels of EVs from alveolar macrophages in BAL of smokers with COPD compared to smokers without COPD and nonsmokers, which correlated with the pack-years and disease severity according to FEV1. Luccheti et al. [57] observed that EVs are detectable in exhaled breathe condensate (EBC) and sputum of COPD patients but did not identify the cell source of these EVs.

**Table 7:** Summary of studies on EVs in COPD diagnosis

Diagnostic				
Author, year [Ref.]	Title	Aim	Type	Conclusion
Lacedonia et al., 2016 [50]	Microparticles in sputum of COPD patients: a potential biomarker of the disease?	Investigate the presence and source of sputum MPs in COPD patients and to correlate the number and source of MPs to the clinical picture	Ex vivo Human	CD31-MPs, CD66b-MPs, and CD235ab-MPs were upregulated in all COPD patients. High levels of CD31-MPs in COPD sputum negatively correlated with FEV1% and could be a new noninvasive method to monitor disease course
Gordon et al., 2011 [51]	Circulating endothelial microparticles as a measure of early lung destruction in cigarette smokers	Evaluate whether plasma EMP levels are elevated in smokers with early lung destruction as assessed by normal spirometry but reduced diffusing capacity of the lung for carbon monoxide (DL_CO_)	Ex vivo Human	Plasma EMPs with apoptotic characteristics are elevated in smokers with normal spirometry but reduced DL_CO_
Takahashi et al., 2014 [52]	Annual FEV1 changes and numbers of circulating endothelial microparticles in patients with COPD: a prospective study	Examine the relationship between EMP number and changes in forced expiratory volume in 1 s (FEV1) in patients with COPD	Ex vivo Human	High E-selectin (CD62E +) EMP level under a stable condition predicted rapid FEV1 decline after a year in patients with COPD. E-selectin EMP number under a stable condition could be a good biomarker to predict the prognosis of patients with COPD
Sundar et al., 2019 [53]	Small RNA-sequence analysis of plasma-derived extracellular vesicle miRNAs in smokers and patients with chronic obstructive pulmonary disease as circulating biomarkers	Investigate whether smoking and progression of chronic lung disease (i.e. COPD) can alter the composition and packaging of proteins, mRNA and ncRNAs in EVs/exosomes	Ex vivo Human In vitro	RNA-seq analysis carried out on EVs from plasma samples of human subjects showed significant miRNAs up- or down-regulated in smokers vs. COPD and non-smokers vs. COPD pairwise comparisons
Jung et al., 2020 [54]	Surface proteome of plasma extracellular vesicles as biomarkers for pneumonia and acute exacerbation of chronic obstructive pulmonary disease	Identify surface proteins of plasma small EVs (ssEVs) as biomarkers for diagnosis and differentiation of AECOPD to CAP (community acquired pneumonia)	Ex vivo Human	There was a significantly higher expression in plasma sEVs (CD45, CD28, CTLA4, TNF-R-II, and CD16) from patients with AECOPD when compared to CAP patients, allowing for discrimination between the two
Koba et al*.*, 2021 [55]	Proteomics of serum extracellular vesicles identifies a novel COPD biomarker, fibulin-3 from elastic fibres	Assess serum EVs to find novel biomarkers for personalised medicine in COPD using the latest proteomic strategies	Ex vivo Human In vivo Mice	This study identified novel biomarkers for COPD using next-generation proteomics of serum extracellular vesicles. Notably, the expression of fibulin-3 is correlated with lung function and emphysema
Soni et al., 2021 [56]	Intra-alveolar neutrophil-derived microvesicles are associated with disease severity in COPD	Evaluate the profiles of intra-alveolar (within BALF) and circulating (within plasma) MVs in COPD patients, characterizing a variety of MV subtype populations	Ex vivo Human	This study identified a variety of MV subtype populations within the BALF and plasma of COPD patients with a spectrum of disease severity. In this heterogeneous patient cohort ranging from mild to very severe COPD, BALF PMN (i.e., neutrophil) MVs strongly correlate with the BODE index as well as multiple other markers of COPD severity: worsening dyspnea score, degree of airway obstruction and hyperinflation, lung parenchymal damage, and exercise tolerance
Lucchetti et al., 2021 [57]	Detection and characterisation of extracellular vesicles in exhaled breath condensate and sputum of COPD and severe asthma patients	Investigate whether extracellular vesicles are present and detectable in exhaled breathe condensate (EBC) and to perform a preliminary comparison of their concentrations in COPD and healthy control subjects	Ex vivo Human	Extracellular vesicles are detectable in EBC and sputum and measurement of EBC mEV concentrations might be more informative in COPD patients
Bazzan et al., 2021 [58]	Microvesicles in bronchoalveolar lavage as a potential biomarker of COPD	investigate the presence and source of MVs in bronchoalveolar lavage (BAL) of smokers with and without COPD compared with nonsmoking controls	Ex vivo Human	MVs obtained directly from the lung BAL show that, in response to smoking and to the development of COPD, measurable inflammatory signals in alveolar macrophages can be quantified and that their numbers are related to the pack-years and the decrease in lung function
Carpi et al., 2020 [59]	Expression analysis of muscle-specific miRNAs in plasma-derived extracellular vesicles from patients with chronic obstructive pulmonary disease	Analyse the expression profiles of EV-derived myo-miRNAs (specifically miR-206, miR-133a-5p, and miR-133a-3p) in plasma samples collected from patients with COPD	Ex vivo Human	Myo-miRNA are present in EV in the plasma of COPD patients and their expression (miR-206, miR-133a-5p, and miR-133a-3p) can discriminate between COPD patients
Shen et al., 2021 [60]	A novel diagnostic signature based on three circulating exosomal mircoRNAs for chronic obstructive pulmonary disease	Evaluate differentially expressed exo-miRNAs in the plasma of patients with COPD and healthy individuals for their potential diagnostic value in COPD	Ex vivo Human	The expression levels of three exo-miRNAs (miR-23a, miR-221 and miR-574) were found to be negatively associated with the forced expiratory volume in the 1st second/forced vital capacity. The three circulating exosomal miRNAs may serve as novel circulating biomarkers for the diagnosis of COPD
Kaur et al., 2021 [61]	Distinct exosomal miRNA Profiles from BALF and lung tissue of COPD and IPF patients	Compare the miRNA population in the BALF and lung-tissue-derived exosomes from healthy non-smokers, healthy smokers, and patients with COPD in several independent cohorts to identify potential biomarkers to determine the extent of any pulmonary damage at an early stage	Ex vivo Human	Next generation sequencing results identified three differentially expressed miRNAs in the BALF and one in the lung-derived exosomes from COPD patients, compared to healthy non-smokers. Of these, miR-122-5p was three- or fivefold downregulated among the lung-tissue-derived exosomes of COPD patients compared to healthy non-smokers and smokers, respectively. The identified lung-specific miRNAs associated with COPD can serve as potential biomarkers or therapeutic targets

Of the studies that observed EV cargo in COPD patients, four studies observed miRNA profiles in EVs and one study observed proteins. Sundar et al. [53] and Kaur et al. [61] observed distinct miRNA profiles in EVs of COPD patients when compared to smokers and/or non-smokers. Carpi et al. [59] noted that miR-206, miR-133a-5p and miR-133a-3p levels can discriminate between COPD patients. Shen et al. [60] observed that expression levels of three exosomal miRNAs were negatively associated with FEV1. Koba et al. [55] study observed that expression of fibulin-3 correlated with lung function and emphysema. Together these studies indicate that EVs can lead to the development of more accurate biomarkers to diagnose COPD and monitor disease progression.

All twelve studies were assessed with scores ranging from 0.50 to 0.86 (Additional file 1: Table S6). Two studies scored 0.86 due to sample population and their techniques for EV isolation and characterisation. Other studies scored low in the areas of study populations, for either size or number of control groups or both, and in their EV isolation method. The least scoring study also received a lower score for the characterisation of EVs, due to no tracking to determine particle size or no visual characterisation of EVs.

## Discussion

Investigating the role of EVs in COPD holds great potential to understand disease pathogenesis and identify biomarkers. The aim of this systematic review was to interpret and present all available research on EVs in the pathogenesis and diagnosis of COPD to identify existing knowledge and support further research within the field.

The studies identified for this review consistently reported significantly elevated levels of EVs in patients with COPD when compared to their control groups, being either healthy smokers or non-smokers. Many of the studies identified the EVs to be from endothelial cells, suggesting damage in the endothelial layer in patients with COPD. Studies have described an association between endothelial dysfunction and COPD and an increase in levels of apoptotic endothelial cells in the lungs of patients with COPD, with further studies demonstrating in animal models the resulting development of emphysema [62, 63]. In addition, endothelial dysfunction has been identified as a possible key mechanism in airflow obstruction and is associated with increased risk of mortality in COPD patients [64]. The studies identified for this review support the hypothesis that endothelial apoptosis is involved in the early developments of emphysema and that endothelial cells increase the release of EVs upon exposure to CS.

Other cell types also release increased levels of EVs during exposure to cigarette smoke. Elevated levels of EVs originated from lung epithelial cells, neutrophils and T lymphocytes, all cells which have previously been identified in the response to exposure of CS and thus play a role in the pathogenesis of COPD [65]. Of particular interest in these studies was the cargo of the EVs that triggered inflammation and degradation of the extracellular matrix (ECM). Epithelial cells were found to release EVs containing proteins and pro-inflammatory cytokines that drive local and systemic inflammation and that resulted in further recruitment of inflammatory cells, particularly neutrophils. Indeed, neutrophilia is a key feature of COPD where neutrophils have previously been observed to secrete proteases that cause destruction of lung tissue and release mediators that further promote inflammation [66]. The studies included described that neutrophils released EVs containing neutrophil elastase, a serine protease known to degrade the ECM, contributing to tissue destruction and emphysema in COPD [67]. T lymphocytes released EVs that reduced cell viability and induced significant production of inflammatory cytokines IL-6, MCP-1, MCP-9 and TNF-a in human bronchial epithelial cells (HBEs), with decreased levels of anti-inflammatory cytokine IL-10 [29]. IL-6 and TNF-a both have a role in the acute phase response of COPD, and TNF-a particularly is significantly associated with disease progression [68]. Together the studies showed that upon CS exposure, epithelial cells, T lymphocytes and neutrophils release increased levels of EVs containing biomolecules that further enhance the inflammatory response and degrade the ECM, causing lung tissue damage. This mechanism in turn drives the development of emphysema. Furthermore, other cells are important in COPD pathogenesis, however, to date there are limited studies on EVs in COPD and current literature only examines EVs produced by endothelial cells, epithelial cells, T lymphocytes, neutrophils and monocytes.

Furthermore, some studies identified investigated the role of EVs containing microRNAs (miRNAs, mi-R) in the mechanism of COPD. miRNAs are small non-coding RNAs essential to key biological functions with the capacity to regulate tens to hundreds of genes simultaneously [69]. As a result, identifying expressions of miRNAs in COPD will enable better understanding of the mechanism of COPD. The studies reported EVs containing significantly increased levels of miRNAs from cells after exposure to CS and in particular, noted this difference in levels for COPD patients when compared to healthy people. One particular miRNA identified in the studies included is miR-21, a microRNA expressed at increased levels in patients with COPD, and when upregulated, drives excessive autophagy in COPD [70]. In addition, the studies noted that upon uptake of the EVs carrying miR-21, macrophages polarised into M2 phenotype and bronchial fibroblast cells differentiated into myofibroblasts. An enhanced polarisation level of M2 phenotype macrophages has been observed in the lungs of smokers, with even higher levels in COPD patients, linking these cells to the pathogenesis of COPD [71]. EVs from cells exposed to CS were also found to carry increased levels of miR-210 among other microRNAs compared to non-exposed cells. These miRNAs caused increased collagen type I, myofibroblast differentiation, and reduced clearance of dead cells. Further to this, myofibroblasts differentiation results in the reduced lung function observed in COPD patients [72]. As a result, investigation into biomolecules or cargo of EVs is essential as these may affect the recipient cells which in turn play a role in the development of COPD.

Bacteria are known to colonise the lower airways in COPD patients and these pathogens also release EVs [9, 40, 73]. The studies included note that bacterial derived EVs in COPD patients had distinct characteristics when compared to healthy smokers and non-smokers. Furthermore, the exposure of bacterial EVs resulted in neutrophilia and increased inflammation, features that lead to the development of emphysema [66]. COPD patients were also found to have higher anti-dust EV IgG titres in serum when compared to control subjects. Dust EVs typically originate from microorganism and as such, may induce neutrophilic pulmonary inflammation and subsequent emphysema as seen in previous animal experiments [43]. Overall, the studies show that bacterial EVs have a key role in the development of COPD, yet not enough research has been done to date. In addition, the assessment scores of these studies were low, highlighting the need for more thorough research in this area.

Patients with COPD suffer from frequent acute exacerbations that cause significant morbidity and mortality, and thus are necessary to prevent [74]. Currently, the exacerbation of COPD is a contentious area to define, despite the fact that these periods present a significant burden on COPD patients. A broad definition of COPD exacerbation is the worsening of the patient’s conditions, although this may be faulty due to no established clinical markers, signs or symptoms that can identify an exacerbation of the condition [75]. COPD exacerbations is defined clinically as periods of increasing respiratory symptoms including cough, increased sputum volume and purulence, wheezing, increased dyspnoea and/or systemic distress, and where there is a need for antibiotics [74]. The studies here observe elevated EV levels in patients with exacerbated COPD when compared to those with stable COPD. In addition, respiratory infection, whether viral or bacterial, may be responsible for periods of exacerbations, as they drive an increased release of functional EVs which contain pro-inflammatory cytokines IL-1B and IL-18 and result in exacerbated neutrophilia. The immune response to the EVs released by these infectious agents can result in increased inflammation and therefore, cause an exacerbation of COPD [65]. Also, CD62E+, CD31+ and other MPs linked to endothelial cells, monocytes and platelets were significantly elevated in AECOPD patients, indicative of endothelial damage and vascular injury in the lungs during exacerbation periods that lead to increased severity of disease. These observations are based on high quality research papers that clearly defined exacerbation of COPD as episodes of worsening symptoms and airway function beyond normal daily variation that required treatment with antibiotics and/or corticosteroids.

Current diagnosis of COPD in clinical practice depends largely on the presence of chronic airflow limitation, normally assessed by post-bronchodilator spirometry [76]. Studies have shown that EVs may present a possible method for COPD diagnosis and progress monitoring, in addition to enabling identification of exacerbation status. Levels of MPs were found to be elevated in patients with COPD which correlate with a rapid FEV1 decline. EMPs with apoptotic characteristics were also found in increased levels, indicating lung damage and endothelial apoptosis. As a result, EMP levels can be used to measure early lung destruction in healthy smokers with normal FEV1 and also allow for COPD diagnosis and monitoring of disease course. Additionally, specific MV subtype populations have been shown to correlate with Body-mass, airflow Obstruction, Dyspnea and Exercise (BODE) index as well as other markers of COPD severity [56]. Furthermore, levels of miRNAs in EVs were significantly different in COPD patients when compared to smokers and to non-smokers, demonstrating that smoking and progression of COPD alter the miRNA levels in circulating EVs, potentially allowing for EV analysis as an added tool for disease biomarkers. Indeed, specific miRNAs were identified as possible biomarkers for diagnosis COPD and discriminating between COPD patients. As a result, the studies show that EVs would serve as a biomarker that would allow for identification of lung damage and diagnosis and monitoring of COPD. These were poor to good quality research studies, highlighting the need to further investigate EVs as a possible biomarker for COPD diagnosis.

Research in this area is limited, therefore primary studies included in the review were heterogeneous (in vivo, ex vivo, in vitro) and were limited to 43 studies with small samples sizes. Additionally, COPD comprises of emphysema and bronchitis, however, current literature did not observe EVs in bronchitis. Furthermore, methods to characterise and measure EVs are heterogeneous and until recently, guidelines for measuring and characterising EVs were not considered. Future research studies should follow the Minimal Information for Studies of Extracellular Vesicle 2018 (MISEV 2018) guidelines. Importantly, this paper highlights the importance of studying EVs in COPD pathogenesis. This review shows consistent reporting of significantly elevated levels of EVs in patients with COPD and AECOPD and that cargo of the EVs from cigarette smoking trigger mechanisms, such as inflammation, that drive pathogenesis of COPD. The studies here also highlighted EVs as possible biomarkers of lung damage for COPD diagnosis and monitoring of disease course.

In conclusion, the studies together show the limited but good quality research examining the role of EVs in COPD. Therefore, more studies are needed to help better define and provide further insight into the functional characteristics of EV in COPD pathogenesis.

## Supplementary Information

**Additional file 1: Table S1.** Assessment of studies on the mechanism of endothelial extracellular vesicles in COPD. **Table S2.** Assessment of studies on the mechanism of EVs of other cell types in COPD. **Table S3.** Assessment of studies on EVs containing miRNA in COPD. **Table S4.** Assessment of studies on bacterial EVs and COPD. **Table S5.** Assessment of studies on EVs in COPD exacerbation. **Table S6.** Assessment of studies on EVs in COPD diagnosis.

## Data Availability

Not applicable.

## Abbreviations

AECOPD: Acute exacerbation of Chronic Obstructive Pulmonary Disease
ApoBDs: Apoptotic bodies
BAL: Bronchoalveolar lavage
BODE: Body-mass, airflow Obstruction, Dyspnea and Exercise
COPD: Chronic Obstructive Pulmonary Disease
CS: Cigarette smoking
DL_CO_: Diffusing Capacity of the Lungs for Carbon Monoxide
EBC: Exhaled breathe condensate
EEVs: Endothelial EVs
ECM: Extracellular matrix
EMPs: Endothelial microparticles
EVs: Extracellular vesicles
FEV1: Forced expiratory volume in 1 s
GOLD: Global Initiative for Chronic Obstructive Lung Disease
HBECs: Human bronchial epithelial cells
ILVs: Intraluminal vesicles
miRNAs, mi-R: MicroRNAs
MPs: Microparticles
MVBs: Multivesicular bodies
MVs: Microvesicles
NE: Neutrophil elastase
PRISMA: Preferred Reporting Items for Systematic Reviews and Meta-Analyses
PCs: Progenitor cells
TLMPs: T lymphocytes microparticles

## References

[1] Barnes PJ (2015). Chronic obstructive pulmonary disease. Nat Rev Dis Prim.

[2] Zuo L (2014). Interrelated role of cigarette smoking, oxidative stress, and immune response in COPD and corresponding treatments. Am J Physiol Lung Cell Mol Physiol.

[3] Kc R (2018). The role of environmental exposure to non-cigarette smoke in lung disease. Clin Transl Med.

[4] Aggarwal T (2019). Oxidative, inflammatory, genetic, and epigenetic biomarkers associated with chronic obstructive pulmonary disorder. J Cell Physiol.

[5] Barnes PJ (2018). Targeting cytokines to treat asthma and chronic obstructive pulmonary disease. Nat Rev Immunol.

[6] Barnes PJ (2010). Chronic obstructive pulmonary disease: effects beyond the lungs. PLoS Med.

[7] Perera WR (2007). Inflammatory changes, recovery and recurrence at COPD exacerbation. Eur Respir J.

[8] Chen YW, Leung JM, Sin DD (2016). A systematic review of diagnostic biomarkers of COPD exacerbation. PLoS ONE.

[9] Kadota T (2016). Extracellular vesicles in chronic obstructive pulmonary disease. Int J Mol Sci.

[10] El Andaloussi S (2013). Extracellular vesicles: biology and emerging therapeutic opportunities. Nat Rev Drug Discov.

[11] Yanez-Mo M (2015). Biological properties of extracellular vesicles and their physiological functions. J Extracell Vesicles.

[12] van der Pol E (2012). Classification, functions, and clinical relevance of extracellular vesicles. Pharmacol Rev.

[13] Javeed N, Mukhopadhyay D (2017). Exosomes and their role in the micro-/macro-environment: a comprehensive review. J Biomed Res.

[14] Atkin-Smith GK (2017). Isolation of cell type-specific apoptotic bodies by fluorescence-activated cell sorting. Sci Rep.

[15] Yang J (2017). Importance of indoor dust biological ultrafine particles in the pathogenesis of chronic inflammatory lung diseases. Environ Health Toxicol.

[16] Thery C (2018). Minimal information for studies of extracellular vesicles 2018 (MISEV2018): a position statement of the international society for extracellular vesicles and update of the MISEV2014 guidelines. J Extracell Vesicles.

[17] Takahashi T (2013). Differences in the released endothelial microparticle subtypes between human pulmonary microvascular endothelial cells and aortic endothelial cells in vitro. Exp Lung Res.

[18] Strulovici-Barel Y (2016). Persistence of circulating endothelial microparticles in COPD despite smoking cessation. Thorax.

[19] Thomashow MA (2013). Endothelial microparticles in mild chronic obstructive pulmonary disease and emphysema. The multi-ethnic study of atherosclerosis chronic obstructive pulmonary disease study. Am J Respir Crit Care Med.

[20] Garcia-Lucio J (2018). Imbalance between endothelial damage and repair capacity in chronic obstructive pulmonary disease. PLoS ONE.

[21] Barak OF (2017). Disturbed blood flow worsens endothelial dysfunction in moderate-severe chronic obstructive pulmonary disease. Sci Rep.

[22] Liu H (2014). Circulating endothelial microparticles involved in lung function decline in a rat exposed in cigarette smoke maybe from apoptotic pulmonary capillary endothelial cells. J Thorac Dis.

[23] Nieri D (2021). Circulating extracellular vesicles are associated with disease severity and interleukin-6 levels in COPD: a Pilot study. J Clin Med.

[24] Lascano J (2021). Association of systemic endothelial-derived and platelet-derived microparticles with clinical outcomes in chronic obstructive pulmonary disease. Chronic Obstr Pulm Dis.

[25] Benedikter BJ (2017). Cigarette smoke extract induced exosome release is mediated by depletion of exofacial thiols and can be inhibited by thiol-antioxidants. Free Radic Biol Med.

[26] Moon H-G (2014). CCN1 secretion and cleavage regulate the lung epithelial cell functions after cigarette smoke. Am J Physiol Lung Cell Mol Physiol.

[27] Genschmer KR (2019). Activated PMN exosomes: pathogenic entities causing matrix destruction and disease in the lung. Cell.

[28] Feller D (2018). Cigarette smoke-induced pulmonary inflammation becomes systemic by circulating extracellular vesicles containing Wnt5a and inflammatory cytokines. Front Immunol.

[29] Qiu Q (2020). Increased airway T lymphocyte microparticles in chronic obstructive pulmonary disease induces airway epithelial injury. Life Sci.

[30] Zou Y (2021). Release and actions of inflammatory exosomes in pulmonary emphysema: potential therapeutic target of acupuncture. J Inflamm Res.

[31] Wang L (2021). Cigarette smoke extract-treated airway epithelial cells-derived exosomes promote M1 macrophage polarization in chronic obstructive pulmonary disease. Int Immunopharmacol.

[32] Song L, Peng J, Guo X (2021). Exosomal lncRNA TCONS_00064356 derived from injured alveolar epithelial type II cells affects the biological characteristics of mesenchymal stem cells. Life Sci.

[33] Xia H (2022). The aberrant cross-talk of epithelium-macrophages via METTL3-regulated extracellular vesicle miR-93 in smoking-induced emphysema. Cell Biol Toxicol.

[34] Margaroli C (2022). A novel in vivo model for extracellular vesicle-induced emphysema. JCI Insight.

[35] Colombo M, Raposo G, Thery C (2014). Biogenesis, secretion, and intercellular interactions of exosomes and other extracellular vesicles. Annu Rev Cell Dev Biol.

[36] He S (2019). Bronchial epithelial cell extracellular vesicles ameliorate epithelial–mesenchymal transition in COPD pathogenesis by alleviating M2 macrophage polarization. Nanomedicine.

[37] Xu H (2018). Exosomal microRNA-21 derived from bronchial epithelial cells is involved in aberrant epithelium-fibroblast cross-talk in COPD induced by cigarette smoking. Theranostics.

[38] Fujita Y (2015). Suppression of autophagy by extracellular vesicles promotes myofibroblast differentiation in COPD pathogenesis. J Extracell Vesicles.

[39] Serban KA (2016). Structural and functional characterization of endothelial microparticles released by cigarette smoke. Sci Rep.

[40] Kim JH (2015). Gram-negative and Gram-positive bacterial extracellular vesicles. Semin Cell Dev Biol.

[41] Kim YS (2015). Extracellular vesicles derived from Gram-negative bacteria, such as *Escherichia coli*, induce emphysema mainly via IL-17A-mediated neutrophilic inflammation. J Immunol.

[42] Kim HJ (2017). The microbiome of the lung and its extracellular vesicles in nonsmokers, healthy smokers and COPD patients. Exp Mol Med.

[43] Kim YS (2016). IgG sensitization to extracellular vesicles in indoor dust is closely associated with the prevalence of non-eosinophilic asthma, COPD, and lung cancer. Allergy Asthma Immunol Res.

[44] Yang J (2020). Lung disease diagnostic model through IgG sensitization to microbial extracellular vesicles. Allergy Asthma Immunol Res.

[45] Takahashi T (2012). Increased circulating endothelial microparticles in COPD patients: a potential biomarker for COPD exacerbation susceptibility. Thorax.

[46] Eltom S (2014). Respiratory infections cause the release of extracellular vesicles: implications in exacerbation of asthma/COPD. PLoS ONE.

[47] Tan DBA (2017). Elevated levels of circulating exosome in COPD patients are associated with systemic inflammation. Respir Med.

[48] Tokes-Fuzesi M (2018). Role of microparticles derived from monocytes, endothelial cells and platelets in the exacerbation of COPD. Int J Chron Obstruct Pulm Dis.

[49] Mirza S (2018). COPD guidelines: a review of the 2018 GOLD report. Mayo Clin Proc.

[50] Lacedonia D (2016). Microparticles in sputum of COPD patients: a potential biomarker of the disease?. Int J COPD.

[51] Gordon C (2011). Circulating endothelial microparticles as a measure of early lung destruction in cigarette smokers. Am J Respir Crit Care Med.

[52] Takahashi T (2014). Annual FEV1 changes and numbers of circulating endothelial microparticles in patients with COPD: a prospective study. BMJ Open.

[53] Sundar IK, Li D, Rahman I (2019). Small RNA-sequence analysis of plasma-derived extracellular vesicle miRNAs in smokers and patients with chronic obstructive pulmonary disease as circulating biomarkers. J Extracell Vesicles.

[54] Jung AL (2020). Surface proteome of plasma extracellular vesicles as biomarkers for pneumonia and acute exacerbation of chronic obstructive pulmonary disease. J Infect Dis.

[55] Koba T (2021). Proteomics of serum extracellular vesicles identifies a novel COPD biomarker, fibulin-3 from elastic fibres. ERJ Open Res.

[56] Soni S (2021). Intra-alveolar neutrophil-derived microvesicles are associated with disease severity in COPD. Am J Physiol Lung Cell Mol Physiol.

[57] Lucchetti D (2021). Detection and characterisation of extracellular vesicles in exhaled breath condensate and sputum of COPD and severe asthma patients. Eur Respir J.

[58] Bazzan E (2021). Microvesicles in bronchoalveolar lavage as a potential biomarker of COPD. Am J Physiol Lung Cell Mol Physiol.

[59] Carpi S (2020). Expression analysis of muscle-specific mirnas in plasma-derived extracellular vesicles from patients with chronic obstructive pulmonary disease. Diagnostics.

[60] Shen Y (2021). A novel diagnostic signature based on three circulating exosomal mircoRNAs for chronic obstructive pulmonary disease. Exp Ther Med.

[61] Kaur G (2021). Distinct exosomal miRNA profiles from BALF and lung tissue of COPD and IPF patients. Int J Mol Sci.

[62] Green CE, Turner AM (2017). The role of the endothelium in asthma and chronic obstructive pulmonary disease (COPD). Respir Res.

[63] Moro L (2008). Endothelial dysfunction in chronic obstructive pulmonary disease. Angiology.

[64] Clarenbach CF, Sievi NA, Kohler M (2017). Annual progression of endothelial dysfunction in patients with COPD. Respir Med.

[65] Taylor JD (2010). COPD and the response of the lung to tobacco smoke exposure. Pulm Pharmacol Ther.

[66] Singh D (2015). Chronic obstructive pulmonary disease, neutrophils and bacterial infection: a complex web involving IL-17 and IL-22 unravels. EBioMedicine.

[67] Crotty Alexander LE, Shin S, Hwang JH (2015). Inflammatory diseases of the lung induced by conventional cigarette smoke: a review. Chest.

[68] Chen J (2020). Change of serum inflammatory cytokines levels in patients with chronic obstructive pulmonary disease, pneumonia and lung cancer. Technol Cancer Res Treat.

[69] Ezzie ME (2012). Gene expression networks in COPD: microRNA and mRNA regulation. Thorax.

[70] Zeng Z (2018). MicroRNA-21 aggravates chronic obstructive pulmonary disease by promoting autophagy. Exp Lung Res.

[71] He S (2017). Characteristics and potential role of M2 macrophages in COPD. Int J Chron Obstruct Pulmon Dis.

[72] Eapen MS (2017). Airway inflammation in chronic obstructive pulmonary disease (COPD): a true paradox. Expert Rev Respir Med.

[73] Murphy TF, Sethi S, Niederman MS (2000). The role of bacteria in exacerbations of COPD. A constructive view. Chest.

[74] Hill AT (2017). Pulmonary exacerbation in adults with bronchiectasis: a consensus definition for clinical research. Eur Respir J.

[75] Rodriguez-Roisin R (2000). Toward a consensus definition for COPD exacerbations. Chest.

[76] Lange P (2016). Diagnosis, assessment, and phenotyping of COPD: beyond FEV(1). Int J Chron Obstruct Pulm Dis.

