# Supplementary material for: Extracellular vesicles and chronic obstructive pulmonary disease (COPD): a systematic review
Source: Respir Res. 2022 Apr 5;23:82. doi: 10.1186/s12931-022-01984-0 (PMC8985325; doi:10.1186/s12931-022-01984-0)
Supplement: Supplementary file 1 — Additional file 1: Table S1. Assessment of studies on the mechanism of endothelial extracellular vesicles in COPD. Table S2. Assessment of studies on the mechanism of EVs of other cell types in COPD. Table S3. Assessment of studies on EVs containing miRNA in COPD. Table S4. Assessment of studies on bacterial EVs and COPD. Table S5. Assessment of studies on EVs in COPD exacerbation. Table S6. Assessment of studies on EVs in COPD diagnosis. [file 12931_2022_1984_MOESM1_ESM.docx]

Additional file 1

**Table S1. Assessment of studies on the mechanism of endothelial extracellular vesicles in COPD**

| Reference | Study populations  (*ex vivo* human) (n/5) | | COPD definition    (n/2) | EV isolation  (n/3) | EV characterisation  (n/4) | Total  (n/14) | Score |
| --- | --- | --- | --- | --- | --- | --- | --- |
|  | Sample size (n/3) | Control groups (n/2) |  |  |  |  |  |
| Strulovici-Barel et al., 2016 | 3 | 2 | 2 | 1 | 4 | 12/14 | 0.86 |
| Thomashow *et al*., 2013 | 3 | 1 | 1 | 1 | 4 | 10/14 | 0.71 |
| Garcia-Lucio *et al.,* 2018 | 3 | 2 | 2 | 1 | 4 | 12/14 | 0.86 |
| Barak *et al*., 2017 | 1 | 1 | 2 | 1 | 4 | 9/14 | 0.64 |
| Nieri *et al*., 2021 | 2 | 0 | 2 | 1 | 3 | 8/14 | 0.57 |
| Lascano *et al.*, 2021 | 2 | 1 | 2 | 1 | 3 | 9/14 | 0.64 |

**Table S2. Assessment of studies on the mechanism of EVs of other cell types in COPD**

| Reference | Study populations  (*ex vivo* human) (n/5) | | COPD definition    (n/2) | EV isolation  (n/3) | EV characterisation  (n/4) | Total  (n/14) | Score |
| --- | --- | --- | --- | --- | --- | --- | --- |
|  | Sample size (n/3) | Control groups (n/2) |  |  |  |  |  |
| Genschmer *et al.,* 2019 | 1 | 1 | 2 | 2 | 3 | 9/14 | 0.64 |
| Feller *et al.* 2018 | 1 | 1 | 0 | 3 | 4 | 9/14 | 0.64 |
| Qiu *et al*., 2020 | 3 | 1 | 2 | 2 | 3 | 11/14 | 0.79 |

**Table S3. Assessment of studies on EVs containing miRNA in COPD.**

| Reference | Study populations  (*ex vivo* human) (n/5) | | COPD definition    (n/2) | EV isolation  (n/3) | EV characterisation  (n/4) | Total  (n/14) | Score |
| --- | --- | --- | --- | --- | --- | --- | --- |
|  | Sample size (n/3) | Control groups (n/2) |  |  |  |  |  |
| He *et al*., 2019 | 1 | 1 | 2 | 3 | 4 | 11/14 | 0.79 |
| Xu *et al*., 2018 | 3 | 2 | 2 | 3 | 3 | 13/14 | 0.93 |
| Serban *et al*., 2016 | 1 | 1 | 2 | 2 | 3 | 9/14 | 0.64 |

**Table S4. Assessment of studies on bacterial EVs and COPD.**

| Reference | Study populations  (*ex vivo* human) (n/5) | | COPD definition    (n/2) | EV isolation  (n/3) | EV characterisation  (n/4) | Total  (n/14) | Score |
| --- | --- | --- | --- | --- | --- | --- | --- |
|  | Sample size (n/3) | Control groups (n/2) |  |  |  |  |  |
| Kim *et al*. 2017 | 1 | 2 | 1 | 2 | 3 | 9/14 | 0.64 |
| Kim & Choi *et al*., 2016 | 3 | 1 | 1 | 2 | 0 | 7/14 | 0.50 |
| Yang *et al*., 2020 | 3 | 1 | 1 | 2 | 0 | 7/14 | 0.50 |

**Table S5. Assessment of studies on EVs in COPD exacerbation.**

| Reference | Study populations  (*ex vivo* human) (n/5) | | COPD definition    (n/2) | EV isolation  (n/3) | EV characterisation  (n/4) | Total  (n/14) | Score |
| --- | --- | --- | --- | --- | --- | --- | --- |
|  | Sample size (n/3) | Control groups (n/2) |  |  |  |  |  |
| Takahashi *et al.,* 2012 | 3 | 2 | 2 | 1 | 4 | 12/14 | 0.86 |
| Tan *et al*., 2017 | 3 | 2 | 2 | 1 | 4 | 12/14 | 0.86 |
| Tokes-Fuzesi *et al*., 2018 | 2 | 2 | 2 | 1 | 4 | 11/14 | 0.79 |

**Table S6. Assessment of studies on EVs in COPD diagnosis.**

| Reference | Study populations  (*ex vivo* human) (n/5) | | COPD definition  (n/2) | EV isolation  (n/3) | EV characterisation  (n/4) | Total  (n/14) | Score |
| --- | --- | --- | --- | --- | --- | --- | --- |
|  | Sample size (n/3) | Control groups (n/2) |  |  |  |  |  |
| Lacedonia *et al.,* 2016 | 1 | 0 | 2 | 1 | 4 | 8/14 | 0.57 |
| Gordon *et al*., 2011 | 3 | 1 | 1 | 1 | 4 | 10/14 | 0.71 |
| Takahashi *et al*., 2014 | 2 | 0 | 2 | 1 | 4 | 9/14 | 0.64 |
| Sundar *et al*., 2019 | 1 | 2 | 2 | 3 | 3 | 11/14 | 0.79 |
| Jung *et al*., 2020 | 1 | 1 | 2 | 1 | 2 | 7/14 | 0.50 |
| Koba *et al.*, 2021 | 2 | 1 | 2 | 3 | 4 | 12/14 | 0.86 |
| Soni *et al*., 2021 | 2 | 0 | 2 | 1 | 3 | 8/14 | 0.57 |
| Lucchetti *et al*., 2021 | 2 | 1 | 2 | 2 | 4 | 11/14 | 0.79 |
| Bazzan *et al*., 2021 | 1 | 2 | 1 | 1 | 3 | 8/14 | 0.57 |
| Carpi *et al*., 2020 | 2 | 0 | 2 | 1 | 3 | 8/14 | 0.57 |
| Shen *et al*., 2021 | 3 | 1 | 2 | 3 | 3 | 12/14 | 0.86 |
| Kaur *et al*., 2021 | 1 | 2 | 1 | 3 | 4 | 11/14 | 0.79 |
